# Longitudinal associations between built environment characteristics and changes in active commuting

**DOI:** 10.1186/s12889-017-4396-3

**Published:** 2017-05-17

**Authors:** Lin Yang, Simon Griffin, Kay-Tee Khaw, Nick Wareham, Jenna Panter

**Affiliations:** 1 MRC Epidemiology Unit & UKCRC Centre for Diet and Activity Research (CEDAR), University of Cambridge, School of Clinical Medicine, Box 285, Cambridge Biomedical Campus, Cambridge, CB2 0QQ UK; 2 Department of Public Health and Primary Care, School of Clinical Medicine, Box 285, Cambridge Biomedical Campus, Cambridge, CB2 0SR UK

**Keywords:** Travel, Active commuting, Environment, GIS, Longitudinal, EPIC-Norfolk

## Abstract

**Background:**

Few studies have assessed the predictors of changes in commuting. This study investigated the associations between physical environmental characteristics and changes in active commuting.

**Methods:**

Adults from the population-based European Prospective Investigation into Cancer (EPIC)-Norfolk cohort self-reported commuting patterns in 2000 and 2007. Active commuters were defined as those who reported ‘always’ or ‘usually’ walking or cycling to work. Environmental attributes around the home and route were assessed using Geographical Information Systems. Associations between potential environmental predictors and uptake and maintenance of active commuting were modelled using logistic regression, adjusting for age, sex and BMI.

**Results:**

Of the 2757 participants (62% female, median baseline age: 52, IQR: 50–56 years), most were passive commuters at baseline (76%, *n* = 2099) and did not change their usual commute mode over 7 years (82%, *n* = 2277). In multivariable regression models, participants living further from work were less likely to take up active commuting and those living in neighbourhoods with more streetlights were more likely to take up active commuting (both *p* < 0.05). Findings for maintenance were similar: participants living further from work (over 10 km, OR: 0.06; 95% CI: 0.25 to 0.13) and had a main or secondary road on route were more likely to maintain their active commuting (OR: 0.52; 95% CI: 0.28 to 0.98). Those living in neighbourhoods with greater density of employment locations were less likely to maintain their active commuting.

**Conclusions:**

Co-locating residential and employment centres as well as redesigning urban areas to improve safety for pedestrians and cyclists may encourage active commuting. Future evaluative studies should seek to assess the effects of redesigning the built environment on active commuting and physical activity.

**Electronic supplementary material:**

The online version of this article (doi:10.1186/s12889-017-4396-3) contains supplementary material, which is available to authorized users.

## Background

Regular engagement in physical activity is associated with a lower risk of developing cardiovascular disease, diabetes, and some cancers [1, 2]. Walking and cycling are accessible to the majority and several reviews have highlighted the benefits of these activities for cardiovascular disease (for example [3, 4]). Walking or cycling all or part of the way to work offers a comparatively easy way to integrate exercise into daily life [5]. A meta-analysis of 8 prospective studies with follow up periods of between 5 and 20 years suggested that active commuting was associated with an 11% reduction in cardiovascular risk [6]. The analysis included a wide range of outcomes such as mortality, incident coronary heart disease, stroke, hypertension and diabetes. Despite the benefits, only 14% of commuters walked or cycled to work in England and Wales in 2011 [7]. In fact, only 24% of all trips in the UK were made using active modes, which is lower than many other European countries including Demark (31%), Germany (32%) and the Netherlands (47%) [8].

Several reviews suggest that environmental attributes such as population density, mixed land use, street connectivity, aesthetics and safety were associated with adults’ walking and cycling for transport [9–12]. Specifically, short commute distance, high street connectivity, living in an urban area and high road density were associated with higher levels of active commuting [9–12]. Importantly, most existing research is predominantly cross-sectional in nature, limiting the causal inferences that can be made. Whilst intervention studies are warranted, it is unclear specifically which attributes of the physical environment should be targeted. Some longitudinal studies have suggested that participants living in more supportive areas for walking or cycling or who reported improvements in convenience for walking or cycling were more likely to report increases in walking and cycling [13–15] or smaller reductions in walking [16]. Another study has suggested that safety is particularly important determinant of changes in active commuting [17]. However, few of these longitudinal studies use objective measures of the environment [13–17].

Understanding the associations between environmental attributes and maintenance as well as uptake of active commuting is essential in order to inform the design of environmental interventions. We therefore aim to contribute to the limited longitudinal evidence in this area by investigating the environmental attributes associated with uptake and maintenance of active commuting over 7 years in a cohort of commuters.

## Methods

### Study design and population

This study uses data from the European Prospective Investigation into Cancer (EPIC) Norfolk cohort. The methods of recruitment, sampling, and overall sample representativeness have been described in detail elsewhere [18]. Briefly, between 1993 and 1997 25,633 adults aged 40–79 were recruited through participating general practices in the county of Norfolk, UK. The study design was approved by Norwich District Health Authority Ethics Committee and all participants provided written informed consent.

Between January 1998 and October 2000, 15,678 (61%) of the cohort attended a health check and completed the EPIC Physical Activity Questionnaire (EPAQ2), a detailed questionnaire on recreational, occupational, utility and household physical activity [19]. This constituted the baseline for the current analysis. Between October 2006 and February 2007, all participants were re-contacted and invited to complete a follow-up questionnaire.

### Active commuting

At baseline and follow-up, participants were asked to report how often they used four types of travel mode to get to their main job (car, work or public transport, bicycle, or on foot) using the response categories of ‘always’, ‘usually’, ‘occasionally’, and ‘never or rarely’. Those who reported ‘always’ or ‘usually’ travelling to work by bicycle or on foot were classified as active commuters. Furthermore, multi-modal commuters who reported ‘always’ or ‘usually’ travelling to work by car or public transport *and* by bicycle or on foot were classified as ‘active commuters’ in order to capture the active element of their journeys. The remainder were classified as passive commuters. Participants were then classified into one of four groups to describe changes in commute mode: those who switched to active commuting, those who switched to passive commuting, those who remained active commuters and those who remained passive commuters.

### Environmental features

Objective assessments of the environmental characteristics in participant’s neighbourhoods and along their routes to work were computed using Geographical Information Systems (GIS). Participants reported their home and work postcode and these were converted into map locations using Address Layer, a dataset that identifies the centre point for all postcodes in the UK [20]. An area of 800 m around the participants’ home location was defined as the neighbourhood environment, which is approximately a 10-min walk. The shortest route was identified between home and work via a modified street network which included pedestrianised streets and footpaths. Table 1 provides an overview of the methods used to derive these characteristics and the classifications used in analysis. These environmental characteristics have been associated with active commuting in previous cross-sectional analyses in this cohort [21].

**Table 1:** Description and classification of objectively-measured neighbourhood and route environmental characteristics

Environmental features	Description	Classification
Neighbourhood		
Urbanization	Urban–rural classification of participants’ home location	Urban/rural
Road density	Total road lengths divided by neighbourhood area (km/km^2^)	Quartile
Proportion of primary road	Presence of primary road	Yes/No
Density of junction	Number of junctions divided by total neighbourhood area	Quartile
Effective walkable area (EWA)	Total neighbourhood area divided by the potential walkable area^a^	Quartile
Density of pavement	Area of pavements divided by total road length	Quartile
Density of pedestrian infrastructure	Area of pedestrian infrastructure divided by total road length	Quartile
Number of streetlight per 100 m	The number of lights divided by road length per 100 m	Quartile
Household Density	Total number of household in the neighbourhood area	Quartile
Density of employment locations	Number of employment locations divided by area of neighbourhood per m^2^	Quartile
Land use mix	Proportion of each land use squared and summed^b^	Quartile
Socioeconomic deprivation	Population weighted scores for neighbourhood	Quartile
Crime rate	Total crimes per 1000 population in area of residence of year April 2005 – March 2006	Quartile
Density of RTAs	The number of RTAs 2002–2006 per km of roads	Quartile
Density of fatal & serious RTAs	The number of serious and fatal RTAs 2002–2006 per km of roads	Yes/No
Accessible land in neighbourhood	Presence of accessible land (all parts and green spaces) in the neighbourhood	Yes/No
Park in neighbourhood	Presence of parks in the neighbourhood	Yes/No
Green space in neighbourhood	Presence of green spaces in the neighbourhood	Yes/No
Route		
Distance from home to work	The shortest route between home and work identified via the modified street network	<1.5 km
		1.5–4 km
		4-10 km
Route length ratio	Route length divided by the straight line distance between the home and school	Quartile
Main road on route	Presence of primary (A) road as part of route	Yes/No
Secondary road on route	Presence of secondary (B) road as part of route	Yes/No
Main or Secondary road along route	Presence of primary (A) or secondary (B) road as part of route	Yes/No
Number of streetlights along route	The number of streetlights along the route divided by route length per 100 m	Quartile
Route land use mix	Proportion of each land use within 100 m of route squared and summed	Quartile
Density of RTAs along route	The total number of RTAs occurred on route between 2002 and 2005 per km of roads	Quartile
Density of fatal & serious RTAs along route	The number of fatal and serious RTAs occurred on route between 2002 and 2005 per km of roads	Yes/No

*RTA* Road traffic accidents

^a^Total neighbourhood area is the area that can be reached via the street network within 800 m from the home and the potential walkable area is the area generated using a circular buffer with a radius of 800 m from the home

^b^Seventeen different land uses were classified: farmland, woodland, grassland, uncultivated land, other urban, beach, marshland, sea, small settlement, private gardens, parks, residential, commercial, multiple use buildings, other buildings, unclassified buildings and roads. This score is also known as the Herfindahl-Hirschman Index developed by Rodriguez and Song (2005)

### Covariates

All covariates were assessed at the initial health check at the time of recruitment. Participants self-reported their date of birth and trained nurses measured height and weight following a standard clinical protocol during a clinic visit. Body mass index (BMI) was calculated as weight/height^2^ (kg/m^2^) and categorized to under/normal weight (<25 kg/m^2^), overweight (25–30 kg/m^2^), and obese (≥30 kg/m^2^) [22]. Age was categorised as up to 50 years, 50–54 years and 55 years and over. Social class was based on occupation and classified according to the Registrar General’s occupation-based classification scheme [23] (professional, managerial and technical; skilled-manual and non-manual and partly skilled or unskilled). Participants additionally self-reported their marital status (and were classified as married or other), smoking status (current smoker, former smoker or never smoker), and drinking behaviour (and classified as non-drinker, sensible or heavy drinker defined using gender specific cut offs) [24].

### Statistical analysis

Descriptive statistics for participants’ socio-demographic characteristics were summarized. Two separate logistic regression models were run to assess i) the odds of taking up active commuting, compared to taking up passive travel, and ii) the odds of maintaining active commuting compared to taking up passive travel.

In univariate analysis, all potential environmental attributes were tested as predictors. Variables were entered into the multiple logistic regression models if they were significant at *p* < 0.05 in univariate analyses [25]. If two or more variables were correlated at least *r* > 0.55, the variable most strongly associated with the outcome was carried forwards. We assessed the significance of categorical variables by examining tests for trend. In multiple logistic regression models, variables were removed if they did not reach the significance level of *p* < 0.05, one at a time, starting with the variable with the highest *p* value. Very few of the socio-demographic characteristics were associated with uptake or maintenance of active commuting (Additional file 1: Table S1) and in general adjustment for these had a small effect on the associations between environmental characteristics and uptake and maintenance. As a result, we chose to adjust our analyses for age, sex and BMI only. All analyses were performed using Stata version 11.0. Although we hypothesised that distance may moderate the associations between environmental predictors and changes in active commuting, the cell sizes for some variables prevented us from formally testing for interactions. Multi-level modelling was not appropriate here as participants were not sampled through postcodes and behaviour was not clustered.

## Results

### Sample characteristics

Of the 15,678 participants who completed EPAQ2 at baseline, 11,009 also returned a questionnaire at follow-up. For these analyses we excluded those who reported not working at either time point (*n* = 5063), failed to provide information on socio-demographic characteristics at recruitment, failed to provide data on commuting behaviour, or reported long term disability which precluded walking or health conditions (*n* = 3189). This left 2757 participants for analysis. Compared to those excluded from the analysis, those included tended to be younger (52.3 vs 60.6 yrs), married (85.8% vs 83.6%) and have a lower BMI (25.8 vs 26.3 kg/m^2^), and were more likely to report having a professional occupation at baseline (all *p* < 0.03).

Table 2 gives details of the characteristics of the sample and 41% of participants lived in urban areas. As there were no significant differences in changes in commute mode by sex, we analysed the entire sample as one group. The majority of the sample were passive commuters at baseline (76%, *n* = 1866). 8% (*n* = 233) of the sample took up active commuting, whilst 14% maintained their active commuting.

**Table 2:** Descriptive characteristics of participants in the EPIC-Norfolk cohort included in the analyses comparing men and women

	Men	Women	*P*
	*n* = 438	*n* = 705	
Age (in years)			
< 50	116 (26.5%)	266 (37.7%)	<0.001
50–54	172 (39.2%)	264 (37.5%)	
> =55	150 (35.3%)	175 (24.8%)	
BMI (kg/m2)			
Normal weight	150 (34.3%)	383 (54.33)	<0.001
Overweight	243 (55.5%)	211 (31.4%)	
Obese	45 (10.3%)	101 (14.3%)	
Social Class			
Professional	219 (50.0%)	287 (40.7%)	0.008
Skilled	159 (36.3%)	311 (44.1%)	
Partly Skilled/unskilled	60 (13.7%)	107 (15.2%)	
Marital Status			
Not married	34 (7.8%)	129 (18.3%)	<0.001
Married	404 (92.2%)	576 (81.7%)	
Alcohol Consumption			
Non drinker	19 (4.3%)	58 (8.2%)	0.008
Sensible drinker	357 (81.5%)	575 (81.6%)	
Heavy drinker	62 (14.2%)	72 (10.2%)	
Smoking Status			
Never smoke	212 (48.4%)	416 (59.0%)	<0.001
Former smoker	194 (44.3%)	223 (31.6%)	
Current smoker	32 (7.3%)	66 (9.4%)	
Change of commuting^a^			
Non-AC to non-AC	265 (60.5%)	412 (58.4%)	0.450
Non-AC to AC	49 (11.2%)	66 (9.4%)	
AC to AC	86 (19.6%)	163 (23.1%)	
AC to non-AC	38 (8.7%)	64 (9.1%)	

^a^AC if the participant engaged in active commuting on their journey to and from work, non-AC if the participant did not engage in active commuting on their journey to and from work. Data are n (proportion in %) unless specified.

### Environmental predictors of uptake of active commuting

In the overall sample, 18 environmental characteristics predicted uptake of active commuting (*p* < 0.05, Additional file 2: Table S2). Although it is difficult to compare the effect sizes between attributes measured using different scales, measures of street density (e.g. density of junctions and roads), infrastructure for pedestrians (e.g. pavements in the neighbourhood and streetlights along the route), safety (e.g. street lighting), traffic (e.g. road traffic accidents) and density of destinations were all associated with uptake. In final adjusted models, only three significant results remained: those whose routes to work were longer or included a main or secondary road were less likely to take up active commuting (over 10 km OR: 0.04; 95% CI: 0.02 to 0.09; main road: OR: 0.45; 95% CI: 0.25 to 0.79) and those whose routes to work had more streetlights (highest quartile (OR: 3.98; 95% CI: 1.85 to 8.57) were more likely to take up active commuting (Table 3).

**Table 3:** Crude and adjusted odds ratio in simple and multiple logistic regression analyses of the association between neighbourhood and route environment characteristics and change in commuting mode stratified by commuting modes at baseline, with adjustment for age, sex and BMI

	Uptake of active commuting				Maintenance of active commuting			
	Model a	*p*	Model b	*p*	Model a	*p*	Model b	*p*
	OR (95% CI)		OR (95% CI)		OR (95% CI)		OR (95% CI)	
Neighbourhood Environment								
Effective walkable area (EWA) (Reference: Lowest)								
Second quartile					0.69 (0.36–1.31)	0.02	0.62 (0.30–1.29)	0.02
Third Quartile	-	-	-	-	0.85 (0.44–1.63)		0.75 (0.35–1.60)	
Highest	-	-	-	-	0.43 (0.22–0.82)		0.32 (0.15–0.68)	
Route Environment								
Distance from home to work (Reference: <1.5 km)								
1.5–4 km	0.31 (0.19–0.52)	<0.001	0.23 (0.13–0.39)	<0.001	2.94 (1.59–5.42)	<0.001	2.85 (1.45–5.59)	<0.001
4–10 km	0.08 (0.04–0.13)		0.06 (0.03–0.11)		8.34 (4.33–16.10)		8.82 (4.15–18.80)	
Main or secondary road on route (Reference: No)								
Yes	-	-	-	-	2.71 (1.56–4.69)	<0.﻿001	1.97 (1.04–3.73)	0.04
Number of streetlights per 100 m (Reference: Lowest)								
Second quartile	3.54 (1.83–6.86)	<0.001	2.54 (1.24–5.21)	<0.001	-	-	-	-
Third Quartile	2.26 (1.12–4.58)		2.04 (0.95–4.39)		-	-	-	-
Highest	4.77 (2.46–9.24)		5.45 (2.62–11.33)		-	-	-	-

Model a: Univariate associations

Model b: Adjusted for baseline age, sex and BMI as well as other environmental predictors listed

### Environmental predictors of maintenance of active commuting

In general, fewer environmental features were associated with maintenance of active commuting than uptake. Ten environmental features predicted maintenance of active commuting with *p* < 0.05 (Additional file 2, Table S2). In final adjusted models, results indicate that those who lived in areas with a greater density of employment locations were more likely to maintain their active commuting (highest quartile OR: 3.13; 95% CI: 1.48 to 6.64). Those with longer routes to work (over 10 km, OR: 0.06; 95% CI: 0.25 to 0.13) and a main or secondary road on their route were less likely to maintain their active commuting (OR: 0.52; 95% CI: 0.28 to 0.98; Table 3).

## Discussion

### Principal findings

This study provides new evidence on the environmental attributes associated with uptake and maintenance of active commuting in adults. In general, we found that supportive environments predicted uptake of active commuting: those living further from work and who had a main or secondary road on their route were less likely to take up and maintain their active commuting. Those with routes to work with more streetlights were more likely to take up active commuting and those who lived in areas with a higher density of employment locations were more likely to maintain their active commuting.

### Strengths and limitations

To the best of our knowledge, this is one of the few studies to investigate the associations between characteristics of the physical environment and changes in active commuting in adults. We use objective measures of the environment and data on commuting from a well-characterised cohort of working adults living in both urban and rural areas which provides environmental heterogeneity. The GIS data were collected before the follow-up period but we are not aware of any major physical environment changes which occurred during the study period, although we acknowledge that there may have been changes in crime rates, traffic volumes and public transport provision during this time. Our measures also considered features of the neighbourhood and route environment. The former were specific to each individual and did not rely on pre-defined neighbourhood areas. Our assessments of the route environment used the shortest route between home and work and these may not represent the actual routes participants used. We have no information on why residents choose to live in particular residential areas and therefore were unable to control for residential self-selection [26]. We do not know whether work locations changed over time as this information was not collected at both time points and no information was available on the characteristics of workplace environments but these would be valuable additions to future studies aiming to understand the role of the environment in shaping changes in active commuting behaviour.

Uptake and maintenance of active commuting were defined using data on habitual commute mode in 2000 and 2007, and we acknowledge there may have been some changes in active commuting behaviours between these time points which we could not capture. In addition, we had no information on the duration or intensity of activity on the commute. We ensured that those who reported always active commuting, even if it was in combination with passive modes of transport were included as it is known that these short bouts of activity can add up to a substantial amount over the course of a week [27, 28]. One study using an objective assessment of activity found that over the course of a week 80% of the journey time on a walking or cycling journey is spent at moderate or vigorous intensity. Even for an 8 min journey, this could amount to over half the recommended levels of physical activity for adults over the course of a working week [28]. Previous studies [9] suggest that environmental features might be differentially associated with walking and cycling. However, in our sample relatively small numbers of participants had changed their usual commute modes, and hence we were unable to explore any potential differences between walking and cycling. Although the study sample are drawn from Norfolk which is a largely rural county with predominantly White population and the majority of whom own a car [8], our sample of healthy, working adults represents a key target group for health promotion.

### Comparison with the existing literature

In our analyses, distance from home to work demonstrated the strongest association with changes in commuting behaviour, which is in line with previous findings [9–17]. We also found that participants living in neighbourhoods with higher numbers of streetlights were more likely to take up active commuting. Well-lit routes may have lower levels of perceived crime and be perceived as safer places to walk. Interestingly crime rates were significantly associated in univariate models but did not remain significant in maximally adjusted models. As such it may be that perceptions of crime rather than actual levels are more important in determining behaviour. Recent studies suggest that increases in resident’s perceptions of fear of crime were associated with decreases in levels of walking [29] and increases in perceived danger for pedestrians and cyclists were associated with increases in car use [13], although others have found no associations between changes in perceptions of safety and walking [30]. However, the best way to reduce fear of and perceived risk from crime is unknown, and this is likely to be sensitive to time, location and social context [31].

We also found participants living in neighbourhoods with a greater density of employment locations were more likely to maintain their active commuting. These findings suggest that co-locating residential and employment areas together will reduce the distance required to travel and therefore encourage active commuting. Whilst we assessed multicollinearity between micro-environmental variables and larger macro-environmental variables such as urban rural status, we carried forward the one which was most strongly related with our outcomes. Even after adjustment for urban rural status, the associations between environmental characteristics and change in active commuting persisted (results not shown). Although cross-sectional studies have also suggested that factors such as connected street networks and high road density are important influences on behaviour [10–13], other factors may act as a barrier to maintenance. We did not have information on changes family circumstances or changing financial or personal situations which have been shown to influence travel behaviour [32].

### Implications and further research

The low prevalence of active commuting in several developed countries [8] suggests that there is a potential to increase cycling and walking behaviour as means of commuting and our findings suggest avenues for intervention which may be effective and should be the focus of future evaluations. Distance from home to work was the strongest predictor of uptake and maintenance of active commuting and local planners may be able to co-locate new residential developments and workplaces, thus reducing the distances required to travel to work. In addition, promoting the use of existing off-site car parks and encouraging short walking or cycling trips from these sites may also be beneficial in terms of accumulating physical activity [27] and improving well-being [33].

Our findings also suggest that higher levels of streetlight provision were associated with uptake of active commuting. Systematic reviews have found some evidence that increased street lighting may lead to improved road safety [34] and overall reductions in crime [35]. Micro-level infrastructure improvements such as street lighting may promote active commuting; however, the mechanisms between safety concerns and activity should be examined in further research. We suggest that perceptions of environment might mediate the association between objective assessment of the physical environment and changes in active commuting and this should be an avenue for future research.

## Conclusions

In this longitudinal study, we found some support for a limited number of potential determinants of changes in active commuting described in the cross-sectional literature. Macro-level changes, such as the redevelopment or regeneration of whole urban areas as well as micro-scale changes at the street level, such as the street lighting may be effective. The effects of these improvements on behaviour needs to be examined in formal evaluative studies, preferably incorporating psychological factors along with environment factors to examine mechanisms underlying the change and maintenance of commuting behaviour.

## Additional files

Additional file 1: Table S1. Results table for univariate associations between sociodemographic characteristics and uptake and maintenance of active commuting. (DOCX 18 kb)

Additional file 2: Table S2. Description of data: Results table for univariate associations between environmental predictors and uptake and maintenance of active commuting. (DOCX 28 kb)

## Abbreviations

BMI: Body mass index
EPAQ2: EPIC Physical Activity Questionnaire
EPIC: European Prospective Investigation into Cancer
GIS: Geographical Information Systems
