# Correction to: longitudinal associations between built environment characteristics and changes in active commuting

**DOI:** 10.1186/s12889-017-4843-1

**Published:** 2017-10-23

**Authors:** Lin Yang, Simon Griffin, Kay-Tee Khaw, Nick Wareham, Jenna Panter

**Affiliations:** 1 MRC Epidemiology Unit & UKCRC Centre for Diet and Activity Research (CEDAR), University of Cambridge, School of Clinical Medicine, Box 285, Cambridge Biomedical Campus, Cambridge, CB2 0QQ UK; 2 Department of Public Health and Primary Care, School of Clinical Medicine, Box 285, Cambridge Biomedical Campus, Cambridge, CB2 0SR UK

## Correction

Following the publication of this article, Yang et al., [1] it has been brought to our attention that there were errors in the abstract and in Tables 2 and 3. These errors occurred as previous versions of the tables were uploaded to the submission system in the process of revising the paper. These errors relate to the presentation of the results. The results reported in the text of the paper are correct and the conclusions are unaltered. We also noted an error in the abstract.

### 1. Error in Table 2

Table 2 reports the characteristics of a sample. The correct version of Table 2 is presented below.

**Table 2:** Descriptive characteristics of participants in the EPIC-Norfolk cohort included in the analyses

Characteristic	%(*n*)
Age (in years)	
< 50	30.4 (839)
50–54	36.4 (1003)
> =55	33.2 (915)
BMI (kg/m2)	
Normal weight	43.4 (1197)
Overweight	43.6 (1203)
Obese	13.0 (357)
Social Class	
Professional	50.6 (1395)
Skilled	36.1 (996)
Partly Skilled/unskilled	13.3 (366)
Marital Status	
Not married	14.2 (392)
Married	85.8 (2365)
Alcohol Consumption	
Non drinker	6.1 (170)
Sensible drinker	80.1 (2208)
Heavy drinker	13.8 (379)
Smoking Status	
Never smoke	53.5 (1475)
Former smoker	38.5 (1062)
Current smoker	8.0 (220)
Total sample for analysis: *n* = 2757	

### 2. Error in Table 3

We also noted that the results presented in Table 3 are incorrect. The correct version of the table appears below and the results reported in the text match the results presented in this Table.

**Table 3:** Adjusted associations between neighbourhood and route environment characteristics and uptake and maintenance of active commuting

	Switching to active commuting (*n* = 2099)		Maintenance of active commuting (*n* = 658)	
	OR (95% CI)	*p*	OR (95% CI)	*p*
Neighbourhood Environment				
Density of employment locations (Reference: Lowest)				
Second quartile	–		1.19 (0.53, 2.72)	0.001
Third Quartile	–		1.53 (0.70, 3.36)	
Highest	–		3.13 (1.48, 6.64)	
Route Environment				
Distance from home to work (Reference: <2 km)				
v2-10 km	0.11 (0.06, 0.18)	0.001	0.22 (0.12, 0.39)	0.001
Over 10 km	0.04 (0.02, 0.09)		0.06 (0.25, 0.13)	
Main or secondary road on route (Reference: No)				
Yes	0.45 (0.25, 0.79)	0.005	0.52 (0.28, 0.98)	0.042
Number of streetlights per 100 m (Reference: Lowest)				
Second quartile	2.76 (1.29, 5.91)	0.002	–	
Third Quartile	2.02 (0.94, 4.34)		–	
Highest	3.98 (1.85, 8.57)		–	
Total sample for analysis: *n* = 2757.				

### 3. Error in the abstract

Those living in neighbourhoods with greater density of employment locations were more likely to maintain their active commuting.
