# Supplementary material for: Longitudinal associations between built environment characteristics and changes in active commuting
Source: BMC Public Health. 2017 May 17;17:458. doi: 10.1186/s12889-017-4396-3 (PMC5527401; doi:10.1186/s12889-017-4396-3)
Supplement: Supplementary file 1 — Results table for univariate associations between sociodemographic characteristics and uptake and maintenance of active commuting. (DOCX 18 kb) [file 12889_2017_4396_MOESM1_ESM.docx]

**Additional File 1**

**Table A1:** Univariate associations between sociodemographic characteristics and uptake and maintenance of active commuting

| **Socio-demographic**  **characteristics** | **Uptake of active**  **commuting** | | **Maintenance of active commuting** | | |
| --- | --- | --- | --- | --- | --- |
|  | OR (95% CI) | *P* | | OR (95% CI) | *p* |
| Age in years (Reference: <=50)  50 - 55 | 1.0  0.87 (0.62, 1.23) | 0.347 | | 1.0  1.10 (0.75, 1.62) | 0.991 |
| >55 | 1.16 (0.83, 1.62) |  | | 1.06 (0.72, 1.58) |  |
| Sex (Reference: Male) | 1.0 |  | | 1.0 |  |
| Female | 1.02 (0.78, 1.34) | 0.877 | | 1.45 (1.06, 2.00) | 0.021 |
| BMI kg/m^2^ (Reference: under/normal weight)  Overweight | 1.0  1.00 (0.75, 1.34) | 0.472 | | 1.0  0.88 (0.63, 1.24) | 0.498 |
| Obese | 1.21 (0.80, 1.83) |  | | 0.92 (0.56, 1.51) |  |
| Social Class (Reference: Professional)  Skilled | 1.0  0.86 (0.64- 1.15) | 0.316 | | 1.0  1.52 (1.07, 2.16) | 0.018 |
| Partly Skilled/unskilled | 0.50 (0.29 -0.84) | 0.009 | | 1.55 (0.97, 2.46) | 0.066 |
| Marital Status (Reference: Not married)  Married | 1.0  1.21 (0.80, 1.85) | 0.370 | | 1.0  1.10 (0.72, 1.68) | 0.652 |
| Alcohol Consumption (Reference: Non drinker)  Sensible drinker | 1.0  1.32 (0.68, 2.56) | 0.062 | | 1.0  0.90 (0.49, 1.64) | 0.878 |
| Heavy drinker | 1.77 (0.86, 3.67) |  | | 0.92 (0.45, 1.89) |  |
| Smoking Status (Reference: Never smoke)  Former smoker | 1.0  1.05 (0.80-1.40) | 0.700 | | 1.0  0.89 (0.63, 1.24) | 0.481 |
| Current smoker | 0.68 (0.38-1.21) | 0.198 | | 0.97 (0.50, 1.86) | 0.925 |
