# Supplementary material for: Longitudinal associations between built environment characteristics and changes in active commuting
Source: BMC Public Health. 2017 May 17;17:458. doi: 10.1186/s12889-017-4396-3 (PMC5527401; doi:10.1186/s12889-017-4396-3)
Supplement: Supplementary file 2 — Description of data: Results table for univariate associations between environmental predictors and uptake and maintenance of active commuting. (DOCX 28 kb) [file 12889_2017_4396_MOESM2_ESM.docx]

**Table A2:** Univariate associations between environmental predictors and uptake and maintenance of active commuting

|  | | **Uptake of active**  **Commuting** | | **Maintenance of active commuting** | |
| --- | --- | --- | --- | --- | --- |
|  | | OR (95% CI) | p | OR (95% CI) | p |
| **Neighbourhood environment** | |  |  |  |  |
| **Urbanization** (Reference: Urban) | |  |  |  |  |
| Rural | | 0.66 (0.50, 0.87) | 0.003 | 0.85 (0.62, 1.17) | 0.321 |
| **Neighbourhood Road Density** (Reference: Lowest) | |  |  |  |  |
| Second quartile | | 0.90 (0.59, 1.36) | 0.001 | 1.31 (0.77, 2.23) | 0.043 |
| Third Quartile | | 1.40 (0.94, 2.08) |  | 0.95 (0.60, 1.50) |  |
| Highest | | 1.82 (1.23, 2.67) |  | 1.73 (1.09, 2.74) |  |
| **Primary road** (Reference: no) | |  |  |  |  |
| Yes | | 1.30 (0.99, 1.71) | 0.062 | 1.35 (0.99, 1.86) | 0.061 |
| **Density of junction** (Reference: Lowest) | |  |  |  |  |
| Second quartile | | 1.22 (0.80, 1.86) | 0.001 | 1.35 (0.81, 2.26) | 0.321 |
| Third Quartile | | 1.69 (1.13, 2.54) |  | 0.93 (0.59, 1.47) |  |
| Highest | | 1.95 (1.30, 2.92) |  | 1.41 (0.89, 2.21) |  |
| **Effective walkable area (EWA)** (Reference: Lowest) | | |  |  |  |
| Second quartile | | 0.77 (0.51, 1.14) | 0.213 | 1.08 (0.68, 1.73) | 0.273 |
| Third Quartile | | 0.86 (0.59, 1.28) |  | 1.03 (0.64, 1.64) |  |
| Highest | | 1.24 (0.86, 1.81) |  | 1.30 (0.84, 2.01) |  |
| **Density of pavement** (Reference: Lowest) | |  |  |  |  |
| Second quartile | | 0.98 (0.65, 1.48) | 0.001 | 0.78 (0.48, 1.28) | 0.280 |
| Third Quartile | | 1.14 (0.76, 1.70) |  | 1.14 (0.70, 1.87) |  |
| Highest | | 1.85 (1.27, 2.71) |  | 1.12 (0.71, 1.77) |  |
| **Density of pedestrian infrastructure** (Reference: Lowest) | | |  |  |  |
| Second quartile | | 1.09 (0.75, 1.59) | 0.478 | 0.83 (0.52, 1.33) | 0.121 |
| Third Quartile | | 0.74 (0.50, 1.11) |  | 0.62 (0.39, 0.99) |  |
| Highest | | 0.98 (0.67, 1.43) |  | 0.74 (0.46, 1.18) |  |
| **Number of streetlight per 100m** (Reference: Lowest) | | |  |  |  |
| Second quartile | | 1.02 (0.67, 1.54) | 0.001 | 1.07 (0.64, 1.79) | 0.124 |
| Third Quartile | | 1.25 (0.85, 1.84) |  | 1.24 (0.78, 1.99) |  |
| Highest | | 1.93 (1.33, 2.79) |  | 1.37 (0.88, 2.13) |  |
| **Household Density** (Reference: Lowest) | |  |  |  |  |
| Second quartile | | 1.23 (0.81, 1.85) | 0.001 | 1.01 (0.59, 1.71) | 0.297 |
| Third Quartile | | 1.48 (0.99, 2.23) |  | 0.92 (0.57, 1.49) |  |
| Highest | | 2.10 (1.41, 3.12) |  | 1.26 (0.79, 2.00) |  |
| **Density of employment locations** (Reference: Lowest) | | |  |  |  |
| Second quartile | 0.96 (0.65, 1.43) | | 0.006 | 0.96 (0.58, 1.60) | 0.003 |
| Third Quartile | 0.98 (0.66, 1.46) | |  | 1.25 (0.76, 2.05) |  |
| Highest | 1.76 (1.20, 2.57) | |  | 1.77 (1.12, 2.81) |  |
| **Land use mix** (Reference: Lowest) |  | |  |  |  |
| Second quartile | 0.92 (0.63, 1.35) | | 0.288 | 1.42 (0.92, 2.20) | 0.631 |
| Third Quartile | 0.68 (0.45, 1.01) | |  | 0.94 (0.61, 1.46) |  |
| Highest | 0.88 (0.60, 1.29) | |  | 0.99 (0.63, 1.57) |  |
| **Socioeconomic deprivation** (Reference: Lowest) |  | |  |  |  |
| Second quartile | 0.87 (0.58, 1.30) | | 0.013 | 0.99 (0.59, 1.63) | 0.365 |
| Third Quartile | 1.25 (0.85, 1.82) | |  | 1.61 (0.98, 2.65) |  |
| Highest | 1.51 (1.02, 2.21) | |  | 1.14 (0.73, 1.78) |  |
| **Crime rate** (Reference: Lowest) |  | |  |  |  |
| Second quartile | 1.42 (0.95, 2.11) | | 0.007 | 0.83 (0.50, 1.37) | 0.723 |
| Third Quartile | 1.14 (0.77, 1.67) | |  | 0.89 (0.57, 1.40) |  |
| Highest | 1.80 (1.25, 2.60) | |  | 1.05 (0.69, 1.60) |  |
| **Number of RTAs per km** (Reference: Lowest) |  | |  |  |  |
| Second quartile | 1.33 (0.89, 1.97) | | 0.004 | 1.00 (0.59, 1.69) | 0.209 |
| Third Quartile | 1.25 (0.83, 1.87) | |  | 1.14 (0.70, 1.87) |  |
| Highest | 1.88 (1.27, 2.80) | |  | 1.28 (0.81, 2.02) |  |
| **Fatal & serious RTAs** (Reference: No) |  | |  |  |  |
| Yes | 1.41 (1.05, 1.89) | | 0.024 | 0.93 (0.64, 1.36) | 0.710 |
| **Accessible land in neighbourhood** (Reference: No) | | |  |  |  |
| Yes | | 1.14 (0.87, 1.50) | 0.337 | 1.39 (1.01, 1.91) | 0.044 |
| **Park in neighbourhood** (Reference: No) | |  |  |  |  |
| Yes | | 1.30 (0.96, 1.74) | 0.086 | 1.33 (0.95, 1.84) | 0.094 |
| **Green space in neighbourhood** (Reference: No) | |  |  |  |  |
| Yes | | 1.12 (0.83, 1.53) | 0.451 | 1.04 (0.75, 1.46) | 0.803 |
| **Route environment** | |  |  |  |  |
| **Route length** (Reference: <2 km) | |  |  |  |  |
| 2.01 – 10km | | 0.31 (0.19, 0.52) | 0.001 | 0.20 (0.12, 0.34) | 0.001 |
| Over 10 km | | 0.12 (0.08, 0.18) |  | 0.19 (0.12, 0.31) |  |
| **Route length ratio** (Reference: Lowest) | |  |  |  |  |
| Second quartile | | 0.99 (0.54, 1.80) | 0.001 | 0.83 (0.44, 1.54) | 0.004 |
| Third Quartile | | 1.50 (0.87, 2.62) |  | 0.81 (0.44, 1.49) |  |
| Highest | | 3.35 (1.99, 5.63) |  | 2.01 (1.15, 3.51) |  |
| **Primary road on route** (Reference: No) | |  |  |  |  |
| Yes | | 0.55 (0.40, 0.76) | 0.001 | 0.43 (0.29, 0.63) | 0.001 |
| **Secondary road on route** (Reference: No) | |  |  |  |  |
| Yes | | 0.77 (0.58, 1.03) | 0.081 | 0.51 (0.36, 0.72) | 0.001 |
| **Primary/Secondary road on route** (Reference: No) | |  |  |  |  |
| Yes | | 0.24 (0.15, 0.37) | 0.001 | 0.27 (0.16, 0.46) | 0.001 |
| **The number of streetlights per 100m** (Reference: Lowest) | |  |  |  |  |
| Second quartile | | 2.41 (1.24, 4.68) | 0.001 | 0.93 (0.48, 1.81) | 0.004 |
| Third Quartile | | 3.01 (1.56, 5.82) |  | 1.03 (0.57, 1.83) |  |
| Highest | | 6.24 (3.33, 11.69) |  | 2.12 (1.20, 3.74) |  |
| **Route land use mix** (Reference: Lowest) | |  |  |  |  |
| Second quartile | | 1.39 (0.82, 2.37) | 0.917 | 1.65 (0.96, 2.85) | 0.938 |
| Third Quartile | | 1.79 (1.08, 2.98) |  | 1.65 (0.93, 2.93) |  |
| Highest | | 0.92 (0.52, 1.63) |  | 0.96 (0.55, 1.68) |  |
| **Number of RTAs per /km route** (Reference: Lowest) | |  |  |  |  |
| Second quartile | | 0.45 (0.24, 0.81) | 0.006 | 0.45 (0.24, 0.84) | 0.647 |
| Third Quartile | | 0.95 (0.57, 1.58) |  | 0.47 (0.27, 0.83) |  |
| Highest | | 1.61 (0.99, 2.60) |  | 0.87 (0.52, 1.46) |  |
| **Fatal & serious RTAs on route** (ref: lowest) | |  |  |  |  |
| Highest | | 0.67 (0.50, 0.91) | 0.010 | 0.53 (0.37, 0.76) | 0.001 |

RTA: Road traffic accidents. Unadjusted associations
